# Community Perceptions of Facilitators and Barriers to Post Natal Care access in Rural Laos

**DOI:** 10.1093/eurpub/ckac130.238

**Published:** 2022-10-25

**Authors:** JC Mayangitan, B Poudel, H Gulliver, C Stephenson

**Affiliations:** Swiss Red Cross, Luang Prabang, Laos

## Abstract

**Background:**

The quality of Maternal, Neonatal and Child Health (MNCH) services delivered varies widely in Lao People Democratic Republic. Swiss Red Cross (SRC) provides support to the country to improve the quality of reproductive health services, enhance access, and positively change health behavior through the MNCH2 project. This implementation research was then undertaken aiming to identify factors affecting decision-making of women relative to accessing postnatal care (PNC) and explore opportunities for improving SRC programming.

**Methods:**

From August 2020 to January 2021, 33 in-depth interviews and 6 focus group discussion (FGD) with 54 women were conducted. Women who had given birth in the last six months were purposively selected from several ethnic groups residing in Chomphet and Phonexay Districts in Luang Prabang province. Socio-cultural and behavioral factors affecting women's decision to access PNC were assessed during the interviews and FGD. Additional perceptions were gathered though interviews with the partners, health service providers, village heads, and external project stakeholders.

**Results:**

Traditional practices such as smoking ritual, strict practice of keeping the baby in the house within the first three days, and the treatment and disposal of placenta were identified as the main barrier for women to access PNC. Perceived importance of these traditional practices, however, are affected by family hierarchy especially with older family members insisting on its practice. Economic, road conditions, and transport challenges were also identified as significant barriers.

**Conclusions:**

Traditional practices and family hierarchy, together with physical and economic access limit women's capacity to engage with facility-based postnatal care. Thus, quality outreach with home visits are critical. Gender inclusive health education given not only to pregnant women but to all family members was also identified as critical and is recommended to improve PNC access.

**Key messages:**

- Post natal care remains the most neglected phase in the provision of quality maternal and newborn care.
- Understanding what influences decision making around health care seeking optimised the goal to continue to increase the numbers of women who access a health facility for post natal care in Laos.

